# Early serum miR-1297 is an indicator of poor neurological outcome in patients with aSAH

**DOI:** 10.1042/BSR20180646

**Published:** 2018-11-21

**Authors:** Bin Sheng, Nian-sheng Lai, Yang Yao, Jin Dong, Zhen-bao Li, Xin-tong Zhao, Jia-qiang Liu, Xue-qin Li, Xing-gen Fang

**Affiliations:** 1 Department of Neurosurgery, The First Affiliated Hospital of Wannan Medical College, Wuhu City 241001, China; 2 Department of Neurosurgery, The Second Affiliated Hospital of Wannan Medical College, Wuhu City 41001, China; 3 Department of Nursing, The First Affiliated Hospital of Wannan Medical College, Wuhu City 241001, China; 4 Department of The Central Laboratory, The First Affiliated Hospital of Wannan Medical College, Wuhu City 241001, China; 5 Non-coding RNA Research Center of Wannan Medical College, Wuhu City 241001, China

**Keywords:** Biomarker, MicroRNA, real-time polymerase chain reaction, Subarachnoid hemorrhage, Stroke

## Abstract

**Objective:** MiRNAs are important regulators of translation and have been described as biomarkers of a number of cardiovascular diseases, including stroke. The purpose of the study was to determine expression levels of serum miR-1297 in patients with aneurysmal subarachnoid hemorrhage (aSAH), and to assess whether miR-1297 was the prognostic indicator of aSAH. **Methods:** We treated 128 aSAH patients with endovascular coiling. The World Federation of Neurological Surgeons (WFNS) grades, Hunt–Hess grades, and modified Fisher scores were used to assess aSAH severity. Neurologic outcome was assessed using the Modified Rankin Scale (mRS) at 1-year post-aSAH. Serum was taken at various time points (24, 72, and 168 h, and 14 days). Serum samples from aSAH patients and healthy controls were subjected to reverse transcription (RT) quantitative real-time PCR (RT-qPCR). **Results:** A poor outcome at 1 year was associated with significantly higher levels of miR-1297 value at the four time points, higher WFNS grade, higher Hunt–Hess grade, and higher Fisher score. Serum miR-1297 levels were significantly higher in patients, compared with healthy controls. There were significant correlations of miR-1297 concentrations in serum with severity in aSAH. The AUCs of miR-1297 at the four time points for distinguishing the aSAH patients from healthy controls were 0.80, 0.94, 0.77, and 0.59, respectively. After multivariate logistic regression analysis, only miR-1297 at 24 and 72 h enabled prediction of neurological outcome at 1 year. **Conclusion:** Serum was an independent predictive factor of poor outcome at 1 year following aSAH. This result supports the use of miR-1297 in aSAH to aid determination of prognosis.

## Introduction

Aneurysmal subarachnoid hemorrhage (aSAH) is a serious, life-threatening type of stroke, characterized by bleeding into the meningeal subarachnoid space surrounding the brain. It is associated with approximately 50% mortality, with 10% of patients dying prior to reaching the hospital, and up to 45% dying within 30 days [1]. Despite the fact that preoperative diagnosis, surgical clipping, endovascular treatment, and intensive care have improved in recent years, overall prognosis remains poor, and aSAH remains a serious health problem. One of the major determinants of the neurological status after aSAH are characteristics of the initial hemorrhage during the hospital course, particularly those related to early brain injury (EBI) and early cerebral vasospasms (CVs) [2]. Subsequently, delayed cerebral ischemia (DCI) occurs in approximately 30%, generally because of CVs, correlating with nearly three-fourths of delayed strokes after aSAH [3]. CV is the most significant cause of neurological morbidity, usually occurring 3 days after aSAH, reaching its peaks at 7 days, and continuing for 2–3 weeks [4]. Clinical trials have demonstrated that the use of an anti-vasospasm drug reduced cerebral angiospasm. Most therapies have been directed at altering the molecular pathways of macrovascular vasospasm. However, clinical outcomes have not achieved desired thresholds [5–7]. Therefore, we need to better understand the pathophysiologic mechanisms of the very early phase of aSAH, such as changes in microvascular filling defects, breakdown of ionic homeostasis, inflammation, and micro-arterial narrowing [8,9]. In addition, we need a reliable, early, cost-effective, and non-invasive approach to screen patients in order to improve prognosis in aSAH.

MicroRNAs (miRNAs) are an abundant class of highly conserved non-coding RNA molecules, consisting approximately 22 nts. They play key roles in regulating translation of mRNAs. Previous studies have shown that levels of various miRNAs in the blood are associated with clinical prognosis in patients with ischemic stroke [10,11]. A similar observation demonstrated significant differences in expression of some miRNAs in human cerebrospinal fluid [12,13]. This suggested that circulating serum miRNAs may potentially be used as biomarkers to indicate tissue damage. Despite the increasing literature regarding miRNAs, only a few studies have examined miRNAs as biomarkers in aSAH [13,14].

Identification of an early prognostic predictor is important for management of patients with aSAH. In our previous study [15], we measured serum miRNAs levels from patients at 72 h after the onset of aSAH, and determined those levels were significantly higher compared with healthy controls. This suggested that it was a potential non-invasive indicator for the presence and progression of aSAH. However, we did not clearly define the most appropriate timing for monitoring the indicator. Therefore, the purpose of the present study was to explore the levels of miR-1297 over various time points in post-aSAH patients and determine the optimal prognostic thresholds of the indicator in aSAH.

## Methods

### Study approval

Study participants were recruited from the Department of Neurosurgery of the First Affiliated Hospital of Wannan Medical College, Wuhu City, China. Written informed consent was received from participants or valid proxies (family or a professional not directly involved in the study) prior to inclusion in the study. All procedures were approved by the Research Ethics Committee of Wannan Medical College.

### Study design

The aSAH patients included in the present study were admitted from May 2015 to January 2016. Inclusion criteria were as follows: (i) recent history of aSAH prior to admission (within a maximum of 24 h following the first symptoms), with evidence of bleeding on computed tomography (CT), and presence of an aneurysm on digital subtraction angiography (DSA); (ii) treatment by coiling or surgical clipping within 72 h after onset of symptoms; (iii) at least 1-year follow-up. Exclusion criteria were as follows: (i) aSAH diagnosis not confirmed; (ii) admission later than 24 h after onset of bleeding; (iii) non-aneurysmal SAH; (iv) coiling or surgical clipping later than 72 h after admission; (v) poor prognosis upon admission without any intervention. All patients were admitted to an intensive care unit after initial treatment. Neurological status was accessed using the modified World Federation of Neurological Surgeons (WFNS) grade and Hunt–Hess grade. Radiological severity of aSAH was assessed using the modified Fisher score [16]. Neurologic outcome was assessed using the Modified Rankin Scale (mRS) at 1-year post-aSAH by structured telephonic interview [17]. If no contact was obtained after this procedure, the patient was declared lost to follow-up. At the end of the follow-up period, patients with an mRS score of 0–2 were classified as having a good outcome, and those with mRS score of 3–5 were classified as having a poor outcome.

### Sample processing

The preliminary study involved a total of 552 serum samples: 128 aSAH patients and 40 healthy controls. Serum was taken at various time points (24, 72, and 168 h, and 14 days), and obtained in the fasting state in each healthy control. The blood samples were processed for serum isolation within 2 h after withdrawal using serum separator tubes. Whole blood was left to stand for approximately 30 min at room temperature before being centrifuged at 3000 rpm for 5 min. Serum was divided into aliquots and stored at −80°C for further analysis.

### RNA isolation

MiRNAs were extracted from serum (200 μl) using the miRcute Serum miRNA Isolation Kit (Tiangen Biotech Co., Ltd.; Beijing, China) according to the manufacturer’s instructions. Briefly, lysis solution (900 μl) was added to a volume of serum (200 μl). An external control (CR100-01, Tiangen Biotech, 0.5 μl) for miRNAs was mixed in the serum prior to miRNA extraction. The aqueous phase containing total RNA was extracted with chloroform and transferred to an RNase-Free Spin Column miRelute, where the protein was removed from the bound RNA. miRNAs were finally eluted in RNase-free water (20 μl), quantitated with the NanoDrop 2000 spectrophotometer (NanoDrop Technologies; Houston, TX, U.S.A.) and stored at −80°C. Only samples with A260/A280 ratio between 1.8 and 2.0 were utilized for further analysis.

### Reverse transcription

cDNA was synthesized using the miRcute Plus miRNA First-Strand cDNA Synthesis Kit (Tiangen Biotech) on the DNA Engine Opticon 2 Real-Time Cycler (MJ Research, Inc.; Waltham, MA, U.S.A.). Each reaction mixture utilized 2× miRNA RT Reaction Buffer (10 μl), 2 μl miRNA RT Enzyme Mix, 3 μl total RNA, and 5 μl RNase-Free ddH_2_O to a reaction volume to 20 μl. Then, with the following cycle profile: 42°C for 60 min and 95°C for 3 min. Synthesized cDNA was stored at −20°C for further analysis.

### Reverse transcription quantitative real-time PCR

Real-time PCR was performed using the miRcute Plus miRNA qPCR Detection kit (SYBR Green) on CFX96 Touth™ Real-Time PCR Detection Systems (Bio-Rad Lab, Inc., Hercules, California, U.S.A.), using 0.4 μl forward primer (miR-1297, forward: 5′-CATCCTTGCTATCTGGGTGCT-3′), specific to the external control (CR100-01), 10 μl of 2× miRcute Plus miRNA Premix (with SYBR and ROX), 0.4 μl reverse primer (10 μM), 2 μl cDNA, and 7.2 μl ddH_2_O to a final volume of 20 μl (all from Tiangen Biotech). A total volume of 20 μl per reaction was transferred to 96-well plates and incubated for 15 min at 95°C, followed by five cycles at 94°C for 25 s, 65°C for 30 s, and 72°C for 34 s, then 45 cycles at 94°C for 20 s, 60°C for 34 s. All samples were run in triplicate.

Samples with cycle threshold (*C*_t_) over 30 were regarded as having no expression. The relative miRNA level was expressed as −ΔCt, where the raw data of the target miRNA were normalized to the *C*_t_ of the external control. PCR products were separated by agarose gels to determine the product size, and dissociation curves were used to examine the specificity of the qPCR assay.

### Statistical analysis

All data were analyzed using MedCalc version 15.0.0 (Medcalc Software bvba, Ostend, Belgium). Data were presented as mean ± S.D. The Mann–Whitney U test was used to assess the differences between two groups and the Kruskal–Wallis test for differences amongst more than two groups. The correlations amongst the variables were calculated using Spearman rank correlation coefficient analysis. The receiver operating characteristic (ROC) curves were generated for the combined miRNAs and the clinical parameters (age, smoking, gender, hypertension, and so forth), which were calculated for comprehensive probability using multiple regression amongst cases and controls. For comparison of levels of miR-1297 over time between patients with poor and good outcomes, a generalized linear relationship model with Spearman’s correlation coefficient was used due to non-normal distribution of miR-1297. ROC curves were constructed to determine the optimal thresholds of miR-1297 for aSAH. For univariate analysis of the evaluation criterion, 1-year outcome as assessed using the mRS by creating contingency tables. The WFNS grades had been already described as predictive for neurological outcome. A multivariate logistic regression model was analyzed to determine factors that independently predicted the mRS after adjusting for the risk factors that reached *P*<0.1 in the univariate analyses. In the model, the odds ratios and 95% confidence intervals (CIs) of the predictors were estimated. A *P*-value of <0.05 was considered significant.

## Results

### Patient characteristics and comparative outcomes at 1-year post-aSAH

A total of 128 aSAH patients and 40 health controls completed the follow-up. Patient characteristics are summarized in Table 1, according to 1-year outcome. The cohort consisted of 54 males and 74 females with a median age of 59 years (age range: 36–83 years). Health controls consisted of 20 males and 20 females with median age of 45 years (age range: 26–64 years ). The criterion of health controls were as follows: (i) healthy volunteers; (ii) patients with scalp laceration or brain trauma; (iii) patients with intracranial tumors. At 1-year follow-up, patients with higher grade of WFNS, higher Hunt–Hess grade, and higher score of Fisher had poor outcomes (*P*<0.001).

**Table 1:** Clinicopathological features of aSAH according to 1-year outcome

Clinicopathological parameters	mRS 0–2, *n*=111	mRS 3–5, *n*=17	*P*-value
Sex; Male	47	7	0.93
Sex; Female	64	10	
Age; <59	51	4	0.08
Age; ≥59	60	13	
Hypertension; Yes	42	7	0.79
Hypertension; No	69	10	
Smoking; Yes	40	8	0.38
Smoking; No	71	9	
WFNS Grade; I	50	0	<0.001
WFNS Grade; II	32	0	
WFNS Grade; III	24	5	
WFNS Grade; IV	5	10	
WFNS Grade; V	0	2	
Hunt–Hess Grade; I	44	0	<0.001
Hunt–Hess Grade; II	53	1	
Hunt–Hess Grade; III	13	10	
Hunt–Hess Grade; IV	1	6	
Hunt–Hess Grade; V	0	0	
Fisher Score; I	33	0	<0.001
Fisher Score; II	46	1	
Fisher Score; III	25	4	
Fisher Score; IV	7	12	

The median age was 59 years.

### Serum levels of miR-1297 in aSAH patients and healthy controls

**Figure 1:**
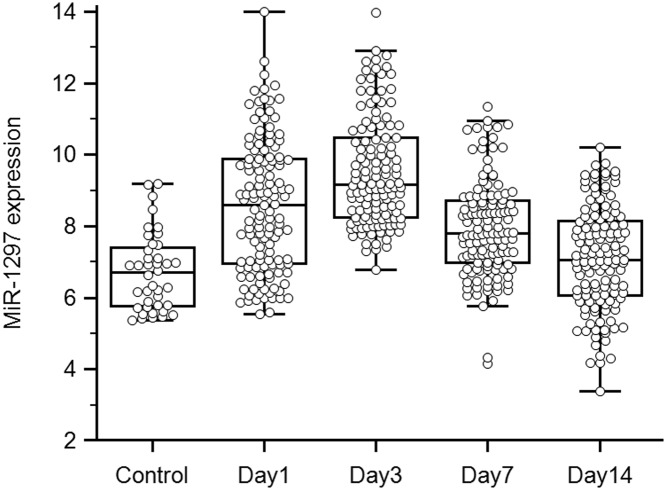
Expression of miR-1297 at four time points in aSAH patients and healthy controls

Expression levels of miR-1297 were determined in 128 aSAH patients and 40 healthy controls. We demonstrated that the serum levels of miR-1297 were moderately higher at 1 day post-aSAH compared with healthy controls, and reached a peak at 7 days. From days 7 to 14, however, we observed a significant decrease in the level of miR-1297 (Figure 1).

### Relationship of miR-1297 with aSAH severity

The WFNS score is a tool used to assess the level of brain injury after aSAH. Higher scores denote greater impairment. Because understanding severity and progression are important for designing future treatments for aSAH patients, the levels of these four time points of miR-1297 were analyzed with respect to various groups classified according to disease severity. At the beginning of the acute period, patients with WFNS grades I–II were classified as mild, and those with grades of III–V were classified as severe.

**Figure 2:**
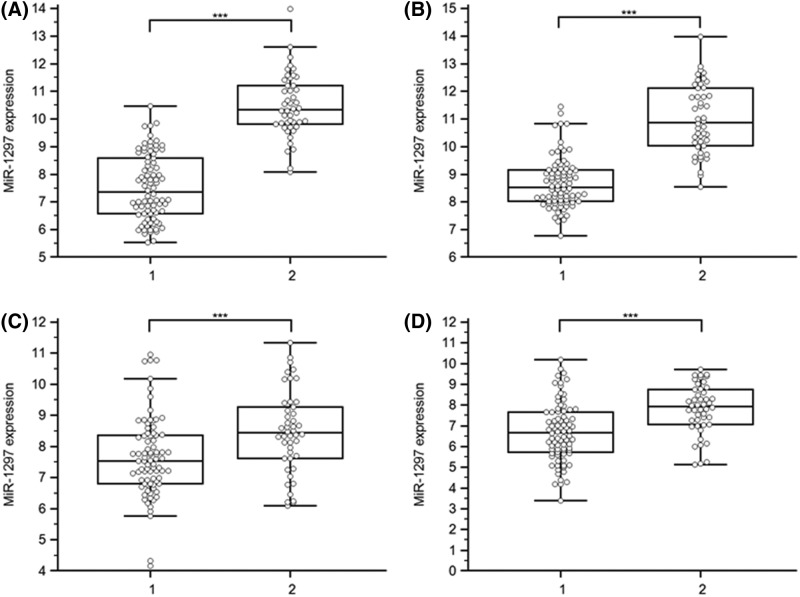
Relative levels of miR-1297 in aSAH patients with severe aSAH (2, WFNS grades III– V) and those with mild aSAH (1, WFNS grades I–II) (**A**–**D**) Serum samples were collected at four time points (24, 72, and 168 h, and 14 days) after SAH and the miRNAs were assayed by qRT-PCR; ***P<0.001. Abbreviation: NS, no significance.

There were some significant differences in the expression of serum miR-1297 at the four time points between severe and mild aSAH (Figure 2). To further investigate the relationships between expression levels and WFNS score at these four time points, we performed Spearman correlation coefficient analysis. In aSAH patients, serum levels of miR-1297 at 24 h (ρ = 0.77; *P*<0.001; 95% CI: 0.69–0.84), 72 h (ρ = 0.72; *P*<0.001; 95% CI: 0.63–0.80), 7th day (ρ = 0.30; *P*<0.001; 95% CI: 0.13–0.45), and 14th day (ρ = 0.39; *P*<0.001; 95% CI: 0.24–0.53) negatively correlated with aSAH severity as scored by WFNS grade.

### ROC analysis of serum miR-1297 levels in aSAH at various time points

**Figure 3:**
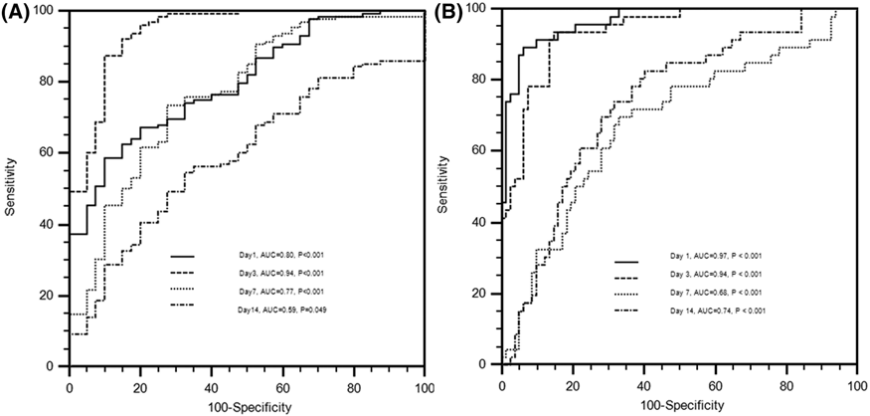
ROC curves to distinguish aSAH patients from healthy controls (**A**) or to distinguish severe from mild aSAH patients (**B**)

Subsequently, the ROC curve was plotted to evaluate the diagnostic accuracy of serum miR-1297 in aSAH. The AUCs of miR-1297 at 24, 72 h, 7, and 14 days for distinguishing the aSAH patients from the controls were 0.80 (95% CI: 0.73–0.86; *P*<0.001), 0.94 (95% CI: 0.90–0.97; *P*<0.001), 0.77 (95% CI: 0.70–0.83; *P*<0.001), and 0.59 (95% CI: 0.52–0.67; *P*=0.049), respectively (Figure 3A). The serum level of miR-1297 at 3 days after aSAH discriminated aSAH patients from healthy controls at other time points with AUC value of 0.94. The sensitivity and specificity at cut-off value of 7.97 were 87.50 and 90%, respectively. These findings suggest that serum miR-1297 had high power to distinguish aSAH patients from healthy controls, especially at 72 h post-aSAH.

To further evaluate the utility of miR-1297 for discrimination of severe aSAH from mild aSAH, we performed ROC curve analysis and found that the AUCs of miR-1297 at 24, 72 h, 7, and 14 days were 0.97 (95% CI: 0.92–0.99; *P*<0.001), 0.94 (95% CI: 0.88–0.97; *P*<0.001), 0.68 (95% CI: 0.59–0.76; *P*<0.001), and 0.74 (95% CI: 0.66–0.81; *P*<0.001), respectively (Figure 3B). Specially, the serum level of miR-1297 at 24 h after aSAH discriminated severe aSAH patients form mild aSAH with AUC value of 0.97.

On univariate analysis, WFNS grade, Hunt–Hess grade, Fisher score, and miR-1297 at 24, 72 h, and 14th day were identified as prognostic predictive factors 1-year post-aSAH (*P*<0.01). In the multivariate logistic regression models, levels of miR-1297 at 24 and 72 h were significantly associated with mRS at 1-year after aSAH (Both *P*<0.01), but not with WFNS grade or Hunt–Hess grade. Association of independent parameters defined AUCs of 0.983 (95% CI: 0.942–0.997) at 24 h, and 0.978 (95% CI: 0.935–0.996) at 72 h. This suggests that severe neurological status on admission and the levels of miR-1297 have high risk of poor outcome (Table 2).

**Table 2:** Multivariable logistic analyses of risk factors in patients with aSAH at 1 year

	Parameter	OR (95% CI)	*P*
Model 1	WFNS grade	1.87 (0.19–18.20)	0.59
	Hunt–Hess grade	4.73 (0.52–43.47)	0.17
	Modified Fisher Score	1.12 (0.25–5.08)	0.89
	miR-1297 levels at day 1	5.81 (1.68–20.11)	0.005
Model 2	WFNS grade	1.02 (0.12–8.92)	0.98
	Hunt–Hess grade	8.54 (0.88–82.44)	0.06
	Modified Fisher Score	1.16 (0.27–4.96)	0.84
	miR-1297 levels at day 3	4.46 (1.50–13.29)	0.007
Model 3	WFNS grade	3.95 (0.65–24.00)	0.14
	Hunt–Hess grade	4.89 (0.69–34.88)	0.11
	Modified Fisher Score	1.42 (0.44–4.65)	0.56
	miR-1297 levels at day 14	0.91 (0.44–1.87)	0.80

Abbreviation: OR, odds ratio.

## Discussion

We developed and validated a novel diagnostic and prognostic tool based on serum miR-1297 to improve the prediction of prognosis for patients with aSAH. Previous studies showed that circulating miRNAs can serve as diagnostic or prognostic biomarkers [18,19]. These studies determined the quantities of miRNAs to serve as diagnosis biomarkers [15]. These reports emphasized the need for more careful evaluation to identify whether miR-1297 levels in the systemic circulation could provide a robust substrate for a non-invasive diagnostic and prognostic strategy.

To our knowledge, the present study is the first to demonstrate the potential role of serum miR-1297 in prediction of EBI in aSAH. Our previous study showed that miR-1297 could be a potential indicator at 72 h post-SAH, but the most appropriate timing for monitoring the indicator was not clearly defined. In the present study, reverse transcription (RT) quantitative real-time PCR (RT-qPCR) assays showed that miR-1297 expression elevated significantly in serum samples of aSAH over various time points compared with healthy controls. Compared with healthy controls, we observed that miR-1297 levels moderately increased from 1 to 3 days, and the increases at 3 days were most pronounced. By contrast, we detected a significant decrease from 3 to 14 days, and the level at 14 days had almost returned to baseline. Multivariate logistic regression associated the level of miR-1297 at 1 and 3 days with a significantly high risk of poor outcome post-aSAH (both *P*<0.01). These results suggest that higher levels of miR-1297 at 24 h (ultra-early stage) and 72 h (early stage) may serve as an indicator of poor neurological prognosis in aSAH patients.

Another interesting finding in our study was that miR-1297 expression in serum also served as a diagnostic indicator for discrimination between severe and mild aSAH patients. It is well known that higher WFNS grade, higher Hunt–Hess grade, and higher Fisher grade correlates with worse clinical outcomes. Levels of miR-1297 were higher in aSAH patients with poor outcomes than those in healthy controls and those with good outcomes. These results suggested that miR-1297 participated in the pathophysiological mechanism of aSAH. Previous studies showed that miR-1297 was involved in many physiological processes via the modulation of gene expression, cell cycle progression, cell proliferation, and signal transduction [20–22]. Yang et al. [23] showed that miR-1297 interacted with Meg3 by regulating growth via PTEN/PI3K/AKT signaling pathway in testicular germ cell tumors. Involvement of miR-1297 has been reported in the deregulation of the glioma. The molecule may prevent proliferation and invasiveness by targetting HMGA1 [20]. A newly described miR-1297/AEG-1/Wnt signaling pathway was studied in prostate cancer [24]. miR-1297 modulated the redox regulation of stem-like cells via targetting xCT in the CD44v–xCT axis in colorectal cancer [25]. Another study showed that miR-1297 played an important role in neural stem cell viability and differentiation via inhibition of Hes1 expression [22]. Another report showed that miR-1297 functioned as an oncogene in regulating the proliferation, cell cycling, and apoptosis in human breast cancer via targetting PTEN/PI3K/AKT signaling [26]. Finally, elevated circulating miR-1297 were biomarkers for the presence and progression of esophageal squamous cell carcinoma and aSAH [15,27]. Taken together, these findings suggest that miR-1297 is associated with disease processes on a molecular level. Based on our previous study of the indicator in aSAH, we hypothesized that higher levels of miR-1297 participated in the development of aSAH.

There are some potential limitations in our study. First, these was a one single-center study with a small number of patients. Larger group studies are needed to validate our results. Moreover, these results cannot be generalized to aSAH patients admitted more than 24 h after the onset of subarachnoid bleeding. Second, the patients underwent surgical treatment or drug therapy prior to the serum samples collected, which may induce changes in expression levels of miR-1297. Third, we evaluated levels of miR-1297 within 14 days following aSAH, however, we cannot monitor these levels continuously everyday. With daily monitoring, we may obtain more accurate timing for prediction of neurological outcome.

Another potential limitation was that we did not routinely perform follow-up CT, but rather followed-up by telephone. Therefore, we could not fully assess recurrent hemorrhage, hydrocephalus, or delayed cerebral ischemia. Finally, the mechanism of miR-1297 as a potentially valuable indicator of prognosis of aSAH remains unknown. Therefore, our next steps will be to explore the pathophysiological mechanisms of miR-1297 in the serum of aSAH patients, which may correlate with gene expression or other biomarkers, such as the exosome.

## Conclusion

In summary, we measured persistent higher levels of miR-1297 after admission in aSAH patients who had worse clinical outcomes. The indicator may be an early independent outcome predictor for patients with aSAH and may aid physicians to adapt the level of therapy accordingly.

## Abbreviations

aSAH: aneurysmal subarachnoid hemorrhage
AUC: area under curve
cDNA: complementary DNA
CI: confidence interval
*C*_t_: cycle threshold
CT: computed tomography
CV: cerebral vasospasm
EBI: early brain injury
miRNA: microRNA
mRS: Modified Rankin Scale
qPCR: quantitative reverse-transcription polymerase chain reaction
ROC: receiver operating characteristic
RT: reverse transcription
WFNS: World Federation of Neurological Surgeons
